# lncRNA-disease association prediction based on latent factor model and projection

**DOI:** 10.1038/s41598-021-99493-5

**Published:** 2021-10-07

**Authors:** Bo Wang, Chao Zhang, Xiao-xin Du, Jian-fei Zhang

**Affiliations:** College of Computer and Control Engineering, Qiqihar University, Qiqihar, 161006 People’s Republic of China

**Keywords:** Computational biology and bioinformatics, Bioinformatics

## Abstract

Computer aided research of lncRNA-disease association is an important way to study the development of lncRNA-disease. The correlation analysis of existing data, the establishment of prediction model, prediction of unknown lncRNA-disease association, can make the biological experiment targeted, improve the accuracy of biological experiment. In this paper, a lncRNA-disease association prediction model based on latent factor model and projection is proposed (LFMP). This method uses lncRNA-miRNA association data and miRNA-disease association data to predict the unknown lncRNA-disease association, so this method does not need lncRNA-disease association data. The simulation results show that under the LOOCV framework, the AUC of LFMP can reach 0.8964. Better than the latest results. Through the case study of lung and colorectal tumors, LFMP can effectively infer the undetected lncRNA-disease association.

## Introduction

lncRNA refers to long non-coding RNAs (lncRNAs) with a length of more than 200 nucleotides. In the past, it was thought that lncRNAs had little effect on gene expression^1^. However, in recent years, studies have shown that lncRNAs are closely related to various human diseases, which has triggered a research upsurge in bioinformatics on the association between lncRNAs and diseases^2^. Studies have shown that lncRNAs are involved in diseases through abnormal sequence^3^ and spatial structure^4^, abnormal expression level^5^ and abnormal interaction with binding proteins^6^, thus affecting human health, including diabetes^7^, cardiovascular disease^8^, and various types of cancer^9^. With the development of computer, big data technology is gradually mature. The application of artificial intelligence technology in the research of associations between lncRNA and diseases can accelerate the discovery of the potent association between lncRNA and diseases, improve the accuracy of biological experiments, and reduce the efforts of bioinformatics researchers and the cost of biological experiments. In medicine, the association between lncRNA-diseases can help doctors improve the detection of early diseases and targeted treatment of some diseases^10^; in biology, the association between lncRNA-diseases can help researchers systematically understand the pathogen nature of complex diseases^11^. Therefore, it is necessary to analyze the existing data through big data technology and establish a prediction model to predict the association between lncRNA-diseases.

At present, lncRNA-disease association prediction model can be roughly divided into two parts. Part of it is based on single association data. For example, Chen et al. proposed a new lncRNA-disease prediction method (LRLSSP)^12^ based on Laplacian regularized least squares and spatial projection. Firstly, by integrating the above information and Gaussian kernel similarity to make up for the lack of semantic similarity of disease, an accurate lncRNA-disease similarity network was reconstructed, and then Laplacian regularized least squares method was used Small two multiplication is used to estimate the association between lncRNA-diseases and solve the problem of lncRNA-disease sparsity. However, this model has some disadvantages, such as requiring a large number of combined data, and relying too much on the known lncRNA-disease association data; in view of Chen et al.’s problem, the models established by the following scholars do not need to rely on Xie et al. proposed a novel prediction method of human lncRNA-disease Association (NCPHLDA)^13^ based on network consistent projection. The model integrates the above information, including lncRNA cosine similarity network and disease cosine similarity network. NCPHLDA has no requirement for parameters and has good prediction performance. However, there are some limitations. If the known lncRNA-disease correlation is small, the prediction results will be biased. In order to solve the problem of insufficient data set of lncRNA-disease association, Zhang et al. constructed a prediction model of lncRNA-disease association based on comprehensive spatial projection fraction (LDAI-ISPS)^14^. In addition, Li et al. proposes a new network consistency prediction lncRNA-disease association model (NCPLDA)^15^. The probability matrix of lncRNA-disease association is calculated by integrating the above information. Then the lncRNA similarity and disease similarity are obtained based on Gaussian kernel similarity. Finally, the lncRNA-disease association score is obtained by combining the disease space projection score and lncRNA space projection score the effect of prediction. The disadvantage is that this method depends on the quality of the data, and the above methods have achieved good prediction results. A hybrid computing framework (SDLDA)^16^ was proposed by Zeng et al. It is a lncRNA-disease association prediction model based on singular value decomposition and deep learning. The model uses singular value decomposition and deep learning to extract the linear and nonlinear features of lncRNA-disease respectively, and combines the linear and nonlinear features to train SDLDA. The combination of linear and nonlinear features can enhance each other to obtain relatively high-quality features, and the connected vectors are used for the association prediction of lncRNA-disease. The performance of the prediction model has been greatly improved. The disadvantage is that it is difficult for SDLDA to determine the parameters. However, biological association information is generally affected by a variety of factors^17^, only through a single data prediction has certain limitations. The other part is to use multiple association data for prediction. Ding et al. Proposed a novel lncRNA-disease association prediction (TPGLDA)^18^. By integrating gene disease association and lncRNA-disease association, we can better describe the heterogeneity of coding non coding gene disease association and effectively identify potential lncRNA-disease association. Fu et al. proposed Matrix factorization-based data fusion for the prediction of lncRNA-disease associations (MFLDA)^19^. In this way, the weights of the data sources and the correlation matrix of the disease can be assigned to the data sources with less weight to break the potential association of lncRNA-disease. The biggest advantage of this model is that it is easy to predict the correlation between different research objects by sorting out a variety of heterogeneous data sources. However, MFLDA is more inclined to study data sparse matrix, and its performance depends on low-quality and unrelated internal relational data sources. Considering the different correlations between the incidence matrix and multiple internal incidence matrices, Wang et al. improved the MFLDA model proposed above, and proposed a model WMFLDA^20^ which decomposes the weighted matrix of multiple relational data. Firstly, the model constructs a heterogeneous network for different types of entities and multiple relational intranets works for the same type of entities. Then the weights are assigned to these networks, and the cooperative low rank matrix is decomposed. Then, the association between lncRNA-diseases was predicted based on the optimized low rank matrix. WMFLDA model can be applied to all kinds of link prediction problems, and can collect data sources among and within relationships. However, this model ignores the different correlations of multiple relational matrices to target prediction tasks. In addition, Liu et al. Proposed a method A Weighted Graph Regularized Collaborative Matrix Factorization Method for Predicting Novel lncRNA-Disease Associations (WGRCMF)^21^. When the known information is insufficient, the performance of the matrix factorization method decreases significantly. The model A Probabilistic Matrix Factorization Method for Identifying lncRNA-disease Associations (PMFLDA)^22^ developed by Xuan et al. Established a new weighted lncRNA-disease association network through three association networks of lncRNA-miRNA, miRNA disease and lncRNA-disease. The KNN algorithm based on disease semantic similarity and lncRNA function similarity is further updated. Finally, the potential lncRNA-disease association is inferred based on probability matrix decomposition. However, this model relies not only on miRNA and lncRNA association data, miRNA-disease association data, but also on lncRNA-disease association data. The above methods use multi-source data to predict the association between lncRNA and disease, but these methods still need the association between lncRNA and disease. However, lncRNA-disease association data are too sparse. In order to solve these problems, a new lncRNA-disease association prediction method LFMP is proposed in this paper. lncRNA-miRNA association data and miRNA-disease association data were used to calculate lncRNA similarity and disease similarity. The lncRNA-disease potential association was constructed through these two data sets. In the absence of known lncRNA-disease association data, the prediction of unknown lncRNA-disease association data is realized. The simulation results show that the AUC of LFMP can reach 0.8964 under the LOOCV framework. Better than the latest results. Through case studies of lung and colorectal tumors, it is proved that LFMP can effectively infer the undetected lncRNA-disease association.

## Results

### Evaluation metrics

In order to evaluate the performance of LFMP model, we used the ROC curve and AUC value generated by Leave One Out Cross Validation (LOOCV) as the evaluation measure, and compared it with other advanced models, namely CFNBC^23^, NBCLDA^24^. Under the framework of LOOCV, we take the association between each lncRNA and the disease one by one as the test set, By comparing the calculated results with the given threshold, we get four evaluation indexes: True Positive (TP), False Positive (FP), True Negative (TN), False Negative (FN).The True Positive Rate (TPR) and False Positive Rate (FPR) were calculated by the following formula:

1 $$TPR = \frac{TP}{{TP + FN}},$$

2 $$FPR = \frac{FP}{{FP + TN}}.$$

AUC is a performance index to measure the performance of the model. When AUC = 1, the model is perfect; When AUC = [0.85, 0.95], the model is excellent. When AUC = [0.7, 0.85], the performance of the model is general.

### Comparison with other methods

**Figure 1:**
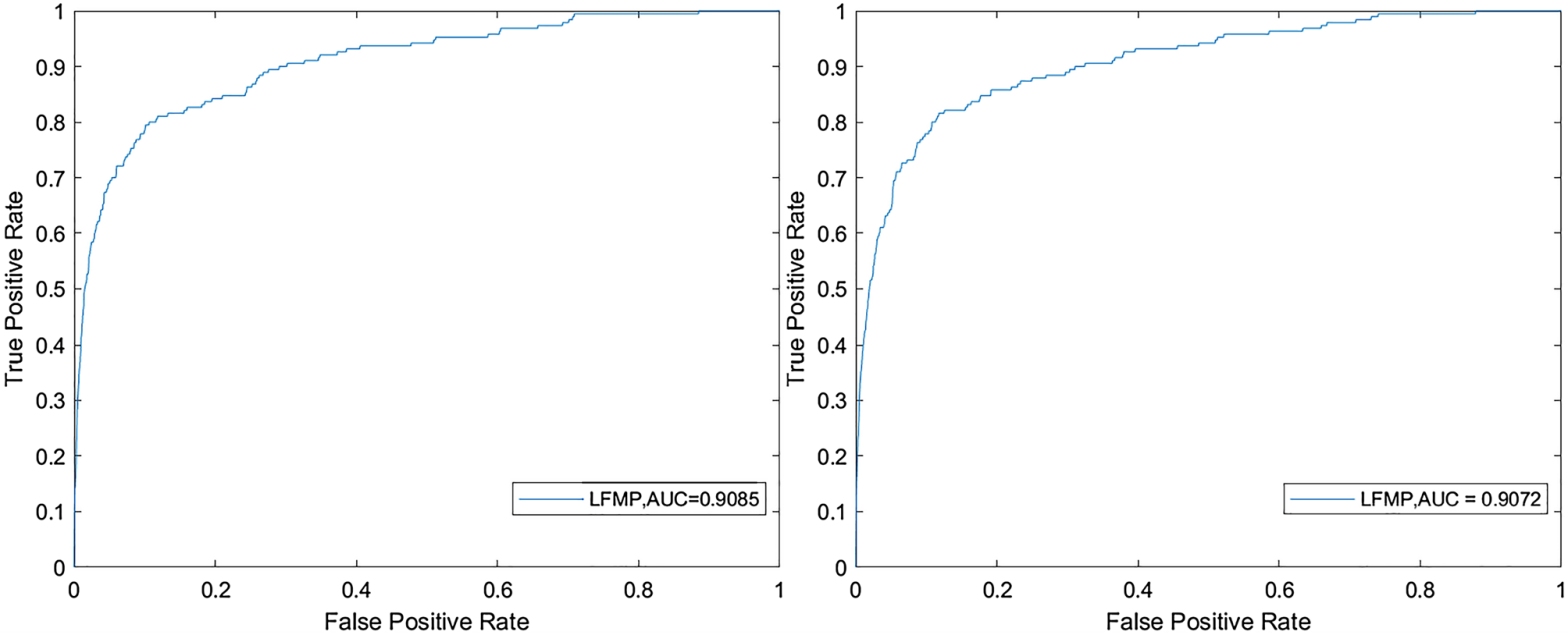
The performance of LFMP in terms of ROC curves and AUC based on 190 known lncRNA-disease associations under the framework of LOOCV frameworks (Left) and fivefold frameworks (Right).

**Figure 2:**
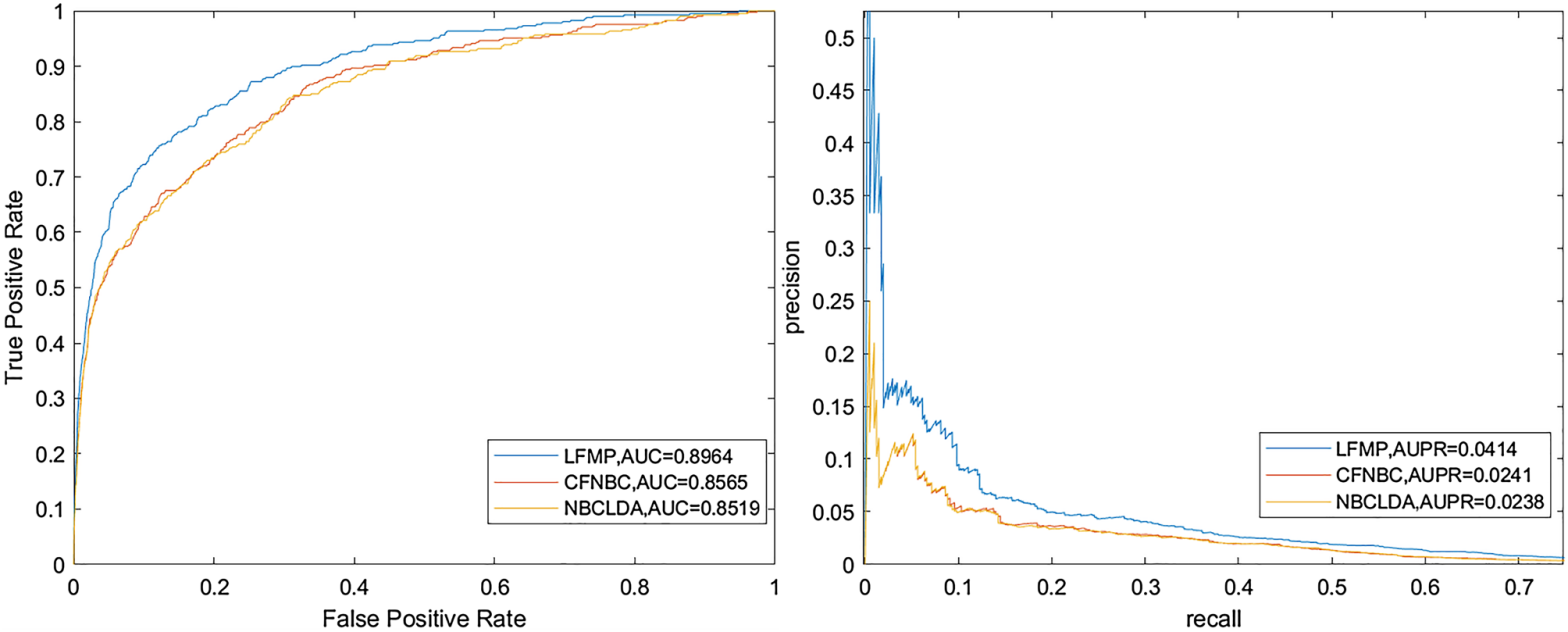
ROC and AUPR comparison between LFMP model and other advanced models based on 407 known lncRNA disease associated LOOCV frameworks.

As shown in the Fig. 1. Based on a 190 known lncRNA-disease associated data set, the AUC values of LFMP under the LOOCV framework and fivefold framework are 0.9085 and 0.9072 respectively. Considering that the biological information used by CFNBC, and NBCLDA is the same as that of LFMP, we compare LFMP with the above three prediction models in the framework of LOOCV. The ROC and AUPR comparison chart based on LOOCV is shown in Fig. 2. It can be seen from ROC comparison chart and AUC value comparison table that CFNBC model is best 0.8576, NBCLDA model is 0.8521, and LFMP model is 0.8964. Obviously, LFMP model is slightly better than other models in ROC curve and AUC value.

### Analysis of parameters

**Figure 3:**
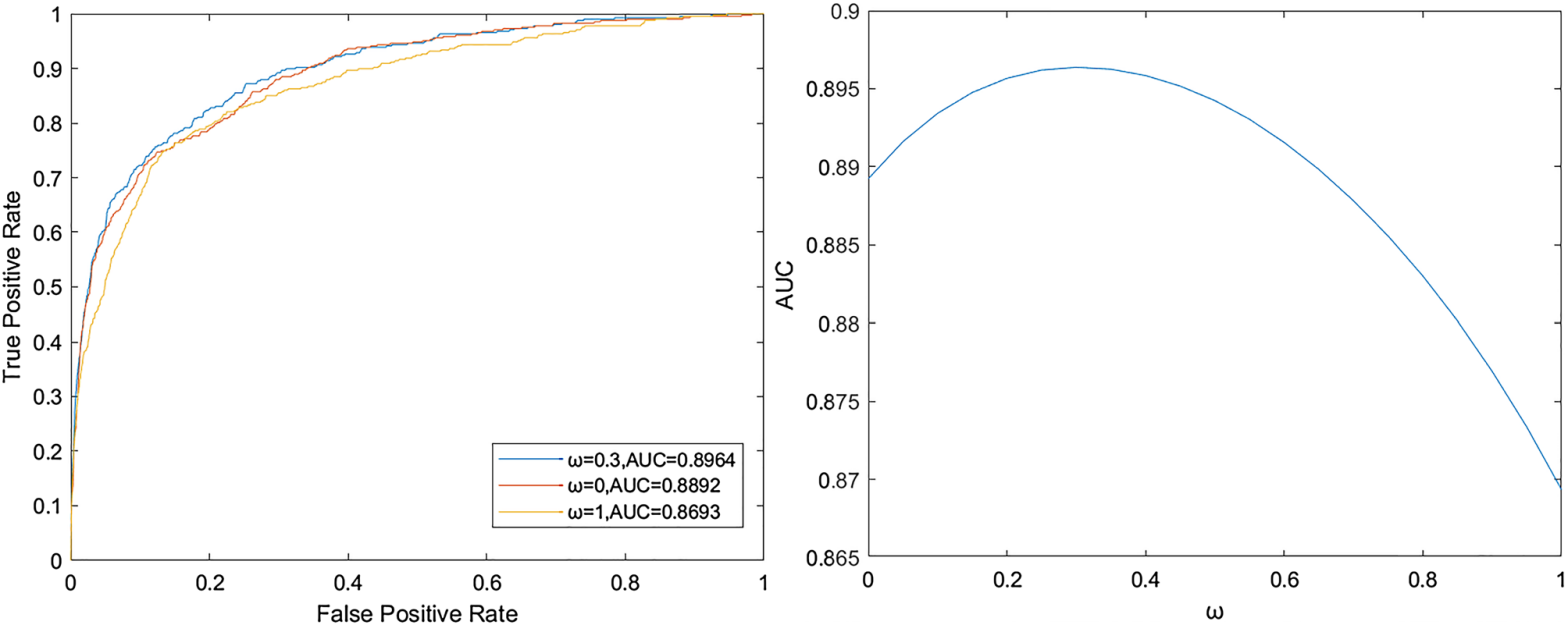
ROC was calculated by lncRNA projection, disease projection and proportional fusion and transformation curve of parameter in the range of [0,1].

In this model, we introduce the parameter ω, whose value range is [0,1]. This parameter is used to adjust the ratio of lncRNA projection fraction and disease projection fraction in the final result calculation. We conducted the experiment with the parameter of 0 and the increment of 0.1, and the results are shown in Fig. 3. It is easy to see that when $\omega$ = 0, only lncRNA-miRNA is used to calculate functional similarity, AUC is 0.8892; when $\omega$ = 1, only lncRNA-disease is used to calculate functional similarity, AUC is 0.8693, while the fused lncRNA similarity matrix is used and the AUC is 0.8964, when $\omega$ = 0.3, which proves that the fusion functional similarity has certain advantages.

### Case studies

In order to further prove LFMP’s potential ability to detect potential lncRNAs associated with diseases, several common diseases were analyzed, and we obtained the rank of related disease prediction through experiments and ranked it. We verified the top 15 lncRNAs by searching the literature, selected the verified lncRNAs and attached the PMID (PMID is the literature number in the fields of life science and medicine included in the PubMed search engine) of relevant supporting literature, as shown in Table 1. Lung cancer (LC) ranks the top three in the world’s cancer incidence rate, ranking the first in cancer death cause in Germany, and the incidence rate of male and female morbidity is 25% and 15% respectively^25^. The original treatment was surgical resection, but not all patients were treated with surgical resection, so the survival rate of patients with lung cancer is very low, about 19%^26^. With the development of bioinformatics, lncRNA, miRNA and other genes have been found to be closely linked with various diseases, and various new lung cancer diagnosis methods and non-surgical treatment methods have emerged, bringing the hope of cure for the majority of lung cancer patients to cure^27,28^. Among the top 15 candidate lncRNAs in our prediction results, 7 lncRNAs have been shown to be associated with lung Neoplasms, in which the lncRNA XIST promote the proliferation and migration of non-small cell lung cancer cells via sponging miR-16 and regulating CDK8 expression^29^; Long Noncoding RNA KCNQ1OT1 Promotes the Progression of Non-Small Cell Lung Cancer via Regulating miR-204-5p/ATG3 Axis^30^; lncRNA NEAT1 Interacted With DNMT1 to Regulate Malignant Phenotype of Cancer Cell and Cytotoxic T Cell Infiltration via Epigenetic Inhibition of p53, cGAS, and STING in Lung Cancer^31^. lncRNA OIP5-AS1 was strongly expressed in lung cancer tissues, which was correlated with tumor size and tumor growth rate. Overexpression of OIP5-AS1 increased the proliferation of lung cancer cells in vitro^32^.

**Table 1:** Candidate lncRNAs and its top 15 cases and related literature.

Disease	lncRNA	Evidence (PMID)	Rank
Lung neoplasms	XIST	31553952	1
Lung neoplasms	KCNQ1OT1	31849486	3
Lung neoplasms	NEAT1	28105699	5
Lung neoplasms	OIP5-AS1	30541307	6
Lung neoplasms	HCG18	32559619	7
Lung neoplasms	SNHG16	31071307	8
Lung neoplasms	FGD5-AS1	33416094	15
Colorectal neoplasms	XIST	33298041	1
Colorectal neoplasms	MALAT1	31311811	3
Colorectal neoplasms	KCNQ1OT1	32564010	4
Colorectal neoplasms	OIP5-AS1	29773344	6
Colorectal neoplasms	NEAT1	30185232	7
Colorectal neoplasms	HCG18	31854468	8
Colorectal neoplasms	DCP1A	29964337	9
Colorectal neoplasms	SNHG16	30962265	10
Colorectal neoplasms	RP4-773N10.5	31966592	14

Colorectal cancer (CRC) is also among the top three cancers in the world, the third most common cancer in men (746,000 cases, 10.0% of the total) and the second most common cancer in women (614,000 cases, 9.2 of the total)^33^. Among the top 15 candidate lncRNAs in our prediction results, 9 have been shown to be associated with colorectal Neoplasms in which MALAT1 polymorphism inhibits the binding of mir-194-5p, leading to the risk, growth and metastasis of colorectal cancer^34^; the long non-coding RNA HCG18 promotes the growth and invasion of colorectal cancer cells through sponging miR-1271 and upregulating MTDH/Wnt/β-catenin^35^; lncRNA MALAT1 promotes the colorectal cancer malignancy by increasing lncRNA DCP1A expression and miR203 downregulation^36^. Long Non-Coding RNA SNHG16 Activates USP22 Expression to Promote Colorectal Cancer Progression by Sponging miR-132-3p^37^.

## Discussion

The research of lncRNA and disease association prediction calculation model has been a hot spot. Using computational models to predict the association between lncRNA and diseases can accelerate the discovery of the potential association between lncRNA and diseases, improve the accuracy of biological experiments, reduce the energy of bioinformatics researchers and the cost of biological experiments, and help doctors improve the early detection and targeted treatment of some diseases. At present, there are a large number of lncRNA-disease prediction models. Most of these models use the association information between lncRNA and disease to predict the unknown lncRNA-disease association, and the most important step to predict the unknown association is the lncRNA-lncRNA similarity calculation and disease-disease similarity calculation. It is commonly used to calculate lncRNA-lncRNA similarity and disease-disease similarity through lncRNA-disease association information. This method has both advantages and disadvantages. The advantage is that the lncRNA-lncRNA calculated directly from the lncRNA-disease association information has more credibility in the prediction of lncRNA-disease association information. However, the disadvantage is that the known lncRNA-disease association information is too sparse, resulting in the lack of known information, which makes the credibility decline. Therefore, we use lncRNA-miRNA association information to calculate lncRNA-lncRNA similarity and miRNA-disease association information to calculate disease-disease similarity. The introduction of miRNA as an intermediate variable makes the credibility of the calculated lncRNA-lncRNA similarity and disease-disease similarity in the prediction of lncRNA-disease association decrease. However, due to the known lncRNA-miRNA association information and miRNA-disease association information are more perfect, the credibility of the calculated lncRNA-lncRNA similarity is improved, Moreover, the introduction of miRNA can solve the problem of lack of lncRNA-disease association information, and provide great help for the prediction of unknown lncRNA-disease association.

## Conclusion

In this study, we propose a lncRNA-disease association prediction model LFMP based on implicit semantic model and projection. The model integrates multiple data, namely lncRNA-miRNA association data and miRNA-disease association data, and realizes indirect prediction of lncRNA-disease association, that is, the model does not need to be based on the known lncRNA-disease association data to predict the association between lncRNA and disease. By comparing with other models and consulting literature to verify the prediction results, it is proved that LFMP has certain reliability and good prediction ability. It is undeniable that our calculation model also has some limitations. Using multivariate data to calculate is a double-edged sword. It helps to improve the reliability of prediction, but also increases the difficulty of obtaining data. Compared with single data association prediction, this model needs more stringent data preprocessing methods, and the model relies too much on the known lncRNA-miRNA association data and miRNA-disease association data. If these two data are too sparse, the prediction performance of the model will be affected.

## Methods

### Dataset and preprocessing

**Figure 4:**
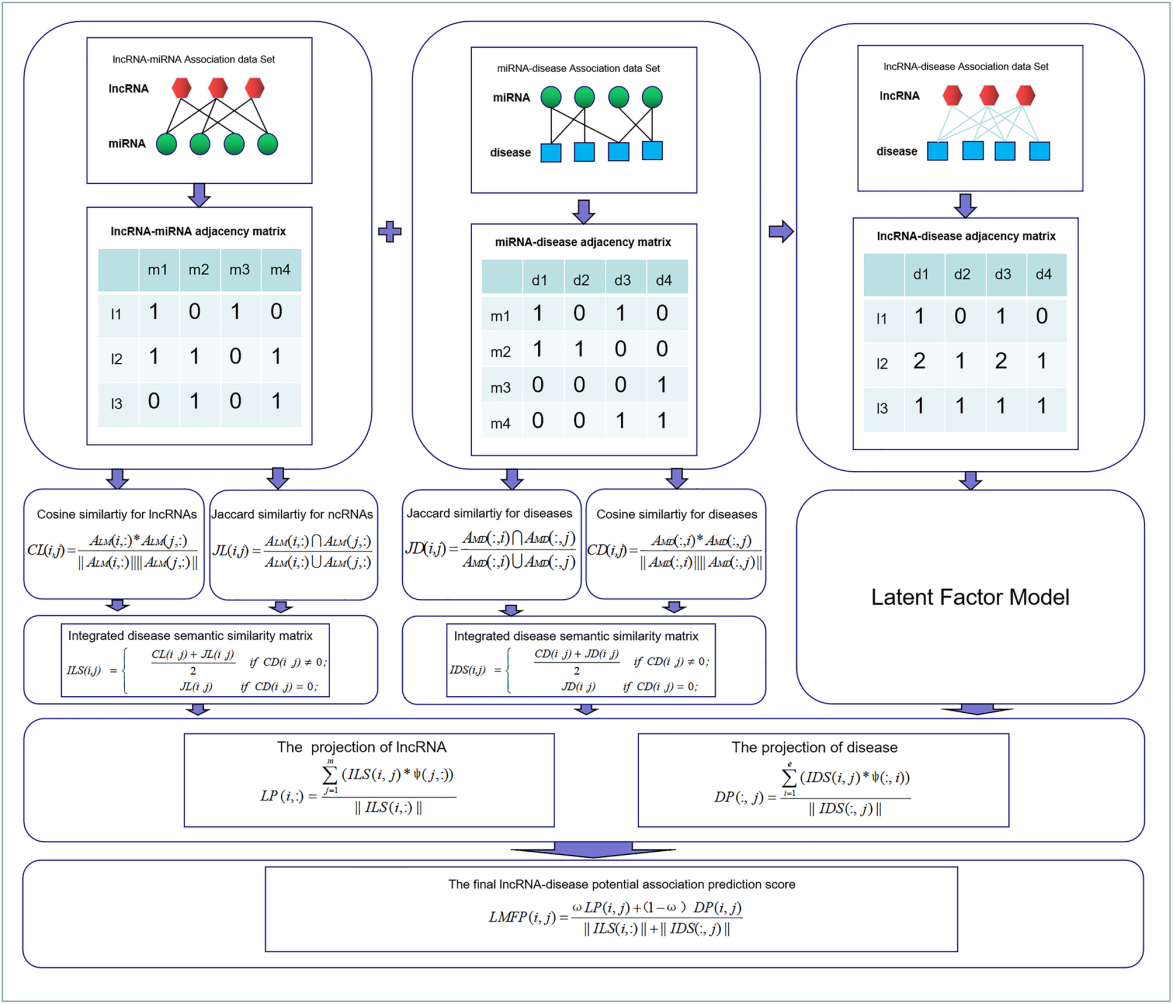
Flow chart of LFMP Applied to lncRNA-disease association prediction.

Download the known lncRNA-disease association datasets from MNDRv2.0 database (2017 Edition)(Supplementary File 3)^38^, Download known miRNA-disease association datasets from HMDD database (2018 Edition)(Supplementary File 1)^39^. Download the known lncRNA-miRNA association datasets from Starbase v2.0 database (2015 Edition)(Supplementary File 2)^40^. The data obtained is cleaned up and the data is finally obtained as shown in Table 1. lncRNA-miRNA adjacency matrix ${\text{A}}_{{{\text{LM}}}} = \{a^{lm} \} m \times n$, miRNA-disease adjacency matrix ${\text{A}}_{{{\text{MD}}}} = \{ a^{md} \} n \times e$ are constructed from lncRNA-miRNA association data set, miRNA-disease association data set. The construction of adjacency matrix is shown in Fig. 4, the experimental data are shown in Table 2.

**Table 2:** List of experimental data.

DATA	lncRNAs	miRNAs	Diseases	Interactions
lncRNA-miRNA	1089	246	–	9086
miRNA-disease	–	246	373	4704
lncRNA-disease	1089	–	373	407
lncRNA-disease	1089	–	373	190

### Cosine similarity for diseases

The cosine similarity for disease between miRNA disease adjacency matrix was calculated:

3 $$CD(i,j) = \frac{A_{MD}(:,i) \times A_{MD}(:,j)}{{||A_{MD}(:,i)||||A_{MD}(:,j)||}},$$

where $A_{MD}(:,i)$ is the i-th column vector in the adjacency matrix of miRNA and disease, which represents the association feature of disease i.

### Jaccard similarity for diseases

The calculation of similarity is an important part of gene association prediction. At present, the methods of similarity calculation in most articles include Gauss interactive calculation of similarity. Compared with the past, we use Jaccard similarity to calculate. The Jaccard similarity for disease between miRNA disease adjacency matrix was calculated:

4 $$JD(i,j) = \frac{A_{MD}(:,i) \cap A_{MD}(:,j)}{{A_{MD}(:,i) \cup A_{MD}(:,j)}}.$$

$AMD(:,i) \cap AMD(:,j)$ is the number of miRNAs associated with disease i and disease j,$A_{MD}(:,i) \cup A_{MD}(:,j)$ is the sum of miRNAs related to disease i and disease j.

### Integrated disease semantic similarity matrix

Integrated disease semantic similarity DS and cosine similarity CD for diseases:

5 $$IDS(i,j) = \left\{ {\begin{array}{*{20}l} {\frac{{CD(i,j) + JD(i,j)}}{2}} \hfill & {if\;CD(i,j) \ne 0;} \hfill \\ {JD(i,j)} \hfill & {if\;CD(i,j) = 0;} \hfill \\ \end{array} } \right..$$

### Cosine similarity for lncRNA

The cosine similarity for lncRNA between lncRNA-miRNA adjacency matrix was calculated:

6 $$CL(i,j) = \frac{A_{LM}(i,:) \times A_{LM}(j,:)}{{||A_{LM}(i,:)||||A_{LM}(j,:)||}}.$$

### Jaccard similarity for lncRNA

The Jaccard similarity for lncRNA between lncRNA-miRNA adjacency matrix was calculated:

7 $$JL(i,j) = \frac{A_{LM}(i,:) \cap A_{LM}(j,:)}{{A_{LM}(i,:) \cup A_{LM}(j,:)}}.$$

### Integrated lncRNA similarity matrix

Integrated miRNA similarity MS and cosine similarity CL for lncRNA:

8 $$ILS(i,j) = \left\{ {\begin{array}{*{20}l} {\frac{{CL(i,j) + JL(i,j)}}{2}} \hfill & {if\;CL(i,j) \ne 0;} \hfill \\ {JL(i,j)} \hfill & {if\;CL(i,j) = 0;} \hfill \\ \end{array} } \right..$$

### Calculation of latent factor model

Compared with previous studies^41,42^, the matrix of lncRNA-disease association was calculated by using the adjacency matrix ${\text{A}}_{{{\text{LM}}}} = {{\{ }}a^{lm} {{\} }}m \times n$ composed of lncRNA-miRNA association information and the adjacency matrix ${\text{A}}_{{{\text{MD}}}} = {{\{ }}a^{md} \} n \times e$ composed of miRNA-disease association information, which was defined as follows:

9 $$A_{LD} = A_{LM} \times A_{MD} .$$

The matrix $A{}_{LD} = \{ a^{ld} \} m \times e$ represents the preliminary correlation score between lncRNA and disease. However, the matrix is still too sparse. In order to solve this problem, we use the latent factor model to calculate the potential score. For matrix $A_{LD} = \{ a^{ld} \} m \times e$, it can be expressed approximately by the product ψ of two matrices X and Y:

10 $$\psi ij = X_{i}^{T} Y_{j} = \sum\limits_{k = 1}^{K} {{\text{x}}_{ik} y_{kj} } .$$

X is the lncRNA feature matrix, Y is the disease feature matrix, and k is an implicit class. X and Y are obtained by $A{}_{LD}$ decomposition, Conversely, the lncRNA feature matrix X is multiplied by the disease feature matrix Y to obtain the lncRNA-disease score matrix ψ (compared with the $A{}_{LD}$ matrix, the ψ matrix has a score for the zero part of the $A{}_{LD}$ matrix, while the corresponding part of the ψ matrix is about equal to $A{}_{LD}$ for the non-zero part of the $A{}_{LD}$ matrix), where in the element in the lncRNA-disease score matrix ψ is the dot product of the corresponding characteristic vector in the matrix X and the matrix Y, It reflects the fit between lncRNA feature and disease feature. Therefore, the larger the number in ψ, the greater the association between lncRNA and disease. In order to obtain the target value, we use the gradient descent method to solve the problem, the loss function is defined as:

11 $$L(X,Y) = \sum\limits_{{\left( {i,j} \right) \in K}} {(\psi ij - X_{i}^{T} Y_{j} } )^{2} + \lambda \sum\limits_{i} {||X_{i} ||^{2} } + \lambda \sum\limits_{j} {||Y_{j} ||^{2} } .$$

Here, $||X_{i} ||$ and $||Y_{j} ||$ are regularization terms used to prevent over fitting, and λ can be obtained experimentally. For each $X_{i}$, the partial derivative is obtained:

12 $$\frac{\partial L}{{\partial X_{i} }} = \frac{{\partial \left[ {\sum\nolimits_{i,j} {\left( {\psi_{ij} - X_{i}^{T} Y_{j} } \right)}^{2} + \lambda \sum\limits_{i} {||X_{i} ||^{2} } } \right]}}{{\partial X_{i} }} = \sum\limits_{j} 2 \left( {X_{i}^{T} Y_{j} - \psi_{ij} } \right)Y_{j} + 2\lambda X_{i} .$$

Then, according to the random gradient descent method, the parameters need to be pushed forward along the fastest descent direction. Therefore, the following recurrence formula can be obtained:

13 $$X_{i} = X_{i} - \alpha \frac{\partial L}{{\partial X_{i} }},$$

where $\alpha$ is the learning rate, Combine formula (12) with formula (13):

14 $$X_{i} = X_{i} - \alpha \sum\nolimits_{j} {2(X_{i}^{T} Y_{j} - \psi ij)Y_{j} + 2\lambda X_{i} } .$$

Similarly, we can get:

15 $$Y_{j} = Y_{j} - \alpha \sum\nolimits_{i} {2(X_{i}^{T} Y_{j} - \psi ij)X_{i} + 2\lambda Y_{j} } .$$

In our experiment, α is set to 0.0002 and λ is set to 0.004.

### Establishment of LFMP prediction model

This paper proposes a new LFMP prediction model by combining the latent factor model and projection. The flow chart of LFMP model is shown in Fig. 4. Compared with previous studies^43^, we further extended the network consistency projection from single lncRNA-disease association data to multivariate data, such as lncRNA-miRNA association data, miRNA-disease association data, and so on. The lncRNA-disease potential score matrix was calculated by the latent factor model. On the lncRNA-disease potential correlation matrix, the functional similarity of the fused lncRNA and the comprehensive disease risk factors were combined the semantic similarity of disease was used to project lncRNA and disease respectively. The projection of lncRNA is defined as:

16 $$LP(i,:) = \frac{{\sum\limits_{j = 1}^{m} {(ILS(i,j) \times \psi (j,:))} }}{||ILS(i,:)||}.$$

In the above formula, $ILS(i,:)$ represents the vector composed of the similarity between lncRNA i and other kinds of lncRNA. $\psi (j,:)$ is potential score matrix between lncRNA j and various diseases. ${||}I{\text{LS}}(i,:){||}$ is the second normal form of vector formed by column i of integrated similarity matrix of lncRNA. $LP(i,j)$ is the projection score. m is the number of lncRNA species. The projection of disease is defined as:

17 $$DP(:,j) = \frac{{\sum\limits_{i = 1}^{e} {(IDS(i,j) \times \psi (:,i)} )}}{||IDS(:,j)||}.$$

$IDS(:,j)$ represents the vector composed of the similarity between disease j and other diseases. $\psi (:,i)$ represents the second normal form of the vector formed by row i of lncRNA-disease potential score matrix. $DP(i,j)$ is the projection score. e is the number of diseases.

The final lncRNA-disease potential association prediction score matrix was formed by fusing lncRNA projection score with disease projection:

18 $$LFMP(i,j) = \frac{{\omega LP(i,j) + \left( {1 - \omega } \right)\;DP(i,j)}}{||ILS(i,:)|| + ||IDS(:,j)||}.$$

$LFMP(i,j)$ is the final association score between lncRNA i and disease j. ω means to regulate lncRNA projection and disease projection in the final result.

## Supplementary Information

Supplementary Legends.

Supplementary Information 1.

Supplementary Information 2.

Supplementary Information 3.

## References

[1] Ponting CP, Oliver PL, Reik W (2009). Evolution and functions of long noncoding RNAs. Cell.

[2] Richard JLC, Eichhorn PJA (2018). Platforms for investigating lncRNA functions. Slas Technol. Transl. Life Sci. Innov..

[3] Li Z, Ma J, Li X, Chan MTV, Wu WKK, Wu Z, Shen J (2019). Aberrantly expressed long non-coding RNAs in air pollution-induced congenital defects. J. Cell Mol. Med..

[4] Ng S-Y, Lin L, Soh BS, Stanton LW (2013). Long noncoding RNAs in development and disease of the central nervous system. Trends Genet..

[5] Sekar S, McDonald J, Cuyugan L, Aldrich J, Kurdoglu A, Adkins J (2015). Alzheimer’s disease is associated with altered expression of genes involved in immune response and mitochondrial processes in astrocytes. Neurobiol. Aging.

[6] Fabrizio FP, Sparaneo A, Trombetta D, Muscarella LA (2018). Epigenetic versus genetic deregulation of the KEAP1/NRF2 axis in solid tumors: Focus on methylation and noncoding RNAs. Oxid. Med. Cell. Longev..

[7] Suwal A, Hao J-L, Liu X-F, Zhou D-D, Pant OP, Gao Y (2019). NONRATT021972 long-noncoding RNA: A promising lncRNA in diabetes-related diseases. Int. J. Med. Sci..

[8] Yan Y, Song D, Song X, Song C (2020). The role of lncRNA MALAT1 in cardiovascular disease. IUBMB Life.

[9] Bhan A, Soleimani M, Mandal SS (2017). Long noncoding RNA and cancer: A new paradigm. Can. Res..

[10] Chi Y, Wang D, Wang J, Yu W, Yang J (2019). Long non-coding RNA in the pathogenesis of cancers. Cells.

[11] Diallo I, Provost P (2020). RNA-sequencing analyses of small bacterial RNAs and their emergence as virulence factors in host-pathogen Interactions. Int. J. Mol. Sci..

[12] Chen M, Peng Y, Li A, Deng Y, Li Z (2020). A novel lncRNA-disease association prediction model using Laplacian regularized least squares and space projection-federated method. IEEE Access.

[13] Xie G, Huang Z, Liu Z, Lin Z, Ma L (2019). NCPHLDA: A novel method for human lncRNA–disease association prediction based on network consistency projection. Mol. Omics.

[14] Zhang Y, Chen M, Li A, Cheng X, Jin H, Liu Y (2020). LDAI-ISPS: lncRNA-disease associations inference based on integrated space projection scores. Int. J. Mol. Sci..

[15] Li G, Luo J, Liang C, Xiao Q, Ding P, Zhang Y (2019). Prediction of lncRNA-disease associations based on network consistency projection. IEEE Access.

[16] Zeng M, Lu C, Zhang F, Li Y, Wu F-X, Li Y, Li M (2020). SDLDA: lncRNA-disease association prediction based on singular value decomposition and deep learning. Methods.

[17] Foffi G, Pastore A, Piazza F, Temussi PA (2013). Macromolecular crowding: Chemistry and physics meet biology (Ascona, Switzerland, 10–14 June 2012). Phys. Biol..

[18] Ding L, Wang M, Sun D, Li A (2018). TPGLDA: Novel prediction of associations between lncRNAs and diseases via lncRNA-disease-gene tripartite graph. Sci. Rep..

[19] Fu G, Wang J, Domeniconi C, Yu G (2018). Matrix factorization-based data fusion for the prediction of lncRNA-disease associations. Bioinformatics.

[20] Wang Y, Yu G, Wang J, Fu G, Guo M, Domeniconi C (2020). Weighted matrix factorization on multi-relational data for lncRNA-disease association prediction. Methods.

[21] Liu J-X, Cui Z, Gao Y-L, Kong X-Z (2021). WGRCMF: A weighted graph regularized collaborative matrix factorization method for predicting novel lncRNA-disease associations. IEEE J. Biomed. Health Inf..

[22] Xuan Z, Li J, Yu J, Feng X, Zhao B, Wang L (2019). A probabilistic matrix factorization method for identifying lncRNA-disease associations. Genes.

[23] Yu J, Xuan Z, Feng X, Zou Q, Wang L (2019). A novel collaborative filtering model for lncRNA-disease association prediction based on the Naïve Bayesian classifier. BMC Bioinform..

[24] Yu J, Ping P, Wang L, Kuang L, Li X, Wu Z (2018). A novel probability model for lncRNA-disease association prediction based on the Naïve Bayesian classifier. Genes.

[25] Wolf U, Barnes B, Bertz J, Haberland J, Laudi A, Stöcker M (2011). Das Zentrum für Krebsregisterdaten (ZfKD) im Robert Koch-Institut (RKI) in Berlin. Bundesgesundh. Gesundh. Gesundh..

[26] Petersen I (2010). Morphologische und molekulare Pathologie des Lungenkarzinoms. Pathologe.

[27] Warth A, Endris V, Kriegsmann M, Stenzinger A, Penzel R, Pfarr N, Weichert W (2015). Molekulardiagnostik des nichtkleinzelligen Lungenkarzinoms. Pathologe.

[28] Wiesweg M, Ting S, Reis H, Worm K, Kasper S, Tewes M (2013). Feasibility of preemptive biomarker profiling for personalised early clinical drug development at a Comprehensive Cancer Center. Eur. J. Cancer.

[29] Zhou X, Xu X, Gao C, Cui Y (2019). XIST promote the proliferation and migration of non-small cell lung cancer cells via sponging miR-16 and regulating CDK8 expression. Am. J. Transl. Res..

[30] Kang Y, Jia Y, Wang Q, Zhao Q, Song M, Ni R, Wang J (2019). Long noncoding RNA KCNQ1OT1 promotes the progression of non-small cell lung cancer via regulating miR-204-5p/ATG3 Axis. Onco. Targets. Ther..

[31] Ma F, Lei Y-Y, Ding M-G, Luo L-H, Xie Y-C, Liu X-L (2020). lncRNA NEAT1 interacted with DNMT1 to regulate malignant phenotype of cancer cell and cytotoxic T cell infiltration via epigenetic inhibition of p53, cGAS, and STING in lung cancer. Front. Genet..

[32] Wang M, Sun X, Yang Y, Jiao W (2018). Long non-coding RNA OIP5-AS1 promotes proliferation of lung cancer cells and leads to poor prognosis by targeting miR-378a-3p. Thoracic Cancer.

[33] Schreuders EH, Ruco A, Rabeneck L, Schoen RE, Sung JJY, Young GP, Kuipers EJ (2015). Colorectal cancer screening: A global overview of existing programmes. Gut.

[34] Wu S, Sun H, Wang Y, Yang X, Meng Q, Yang H (2019). MALAT1 rs664589 polymorphism inhibits binding to miR-194-5p, contributing to colorectal cancer risk, growth, and metastasis. Can. Res..

[35] Li S, Wu T, Zhang D, Sun X, Zhang X (2020). The long non-coding RNA HCG18 promotes the growth and invasion of colorectal cancer cells through sponging miR-1271 and upregulating MTDH/Wnt/β-catenin. Clin. Exp. Pharmacol. Physiol..

[36] Wu C, Zhu X, Tao K, Liu W, Ruan T, Wan W (2018). MALAT1 promotes the colorectal cancer malignancy by increasing DCP1A expression and miR203 downregulation. Mol. Carcinog..

[37] He X, Ma J, Zhang M, Cui J, Yang H (2020). Long non-coding RNA SNHG16 activates USP22 expression to promote colorectal cancer progression by sponging miR-132-3p. OncoTargets Therapy.

[38] Cui T, Zhang L, Huang Y, Yi Y, Tan P, Zhao Y (2017). MNDR v2.0: An updated resource of ncRNA–disease associations in mammals. Nucleic Acids Res..

[39] Li Y, Qiu C, Tu J, Geng B, Yang J, Jiang T, Cui Q (2014). HMDD v2.0: A database for experimentally supported human microRNA and disease associations. Nucleic Acids Res..

[40] Li JH, Liu S, Zhou H, Qu LH, Yang JH (2014). starBase v2.0: Decoding miRNA-ceRNA, miRNA-ncRNA and protein–RNA interaction networks from large-scale CLIP-Seq data. Nucleic Acids Res..

[41] Chen X, Yan G-Y (2013). Novel human lncRNA-disease association inference based on lncRNA expression profiles. Bioinformatics.

[42] Lu C, Yang M, Luo F, Wu F-X, Li M, Pan Y, Li Y, Wang J (2018). Prediction of lncRNA-disease associations based on inductive matrix completion. Bioinformatics.

[43] Chen H, Zhang Z, Li G (2019). Relating disease-gene interaction network with disease-associated NcRNAs. IEEE Access.

