# Supplementary material for: lncRNA-disease association prediction based on latent factor model and projection
Source: Sci Rep. 2021 Oct 7;11:19965. doi: 10.1038/s41598-021-99493-5 (PMC8497550; doi:10.1038/s41598-021-99493-5)
Supplement: Supplementary file 1 — Supplementary Legends. [file 41598_2021_99493_MOESM1_ESM.doc]

Bo Wang1,*, Chao Zhang1, Xiao-xin Du1, and Jian-fei Zhang1

# **lncRNA-Disease Association Prediction Based On Latent Factor Model and Projection**

lncRNA-Disease Association Prediction Based On Latent Factor Model and Projection

lncRNA-Disease Association Prediction Based On Latent Factor Model and Projection

1 College of computer and control engineering,Qiqihar University, Qiqihar 161006, People’s Republic of China

*Correspondence: [drbowang@163.com](../drbowang@163.com)

**Supplementary Information**

**Supplementary file 1.**The human miRNA-disease association dataset was downloaded from HMDD in January, 2018. After getting rid of duplicate associations with the different evidences and merging different miRNA copies which produce the same mature miRNA, this dataset consists of 4704 miRNA–disease associations, including 373 diseases and 246 miRNAs.As shown in Figure 1.

**
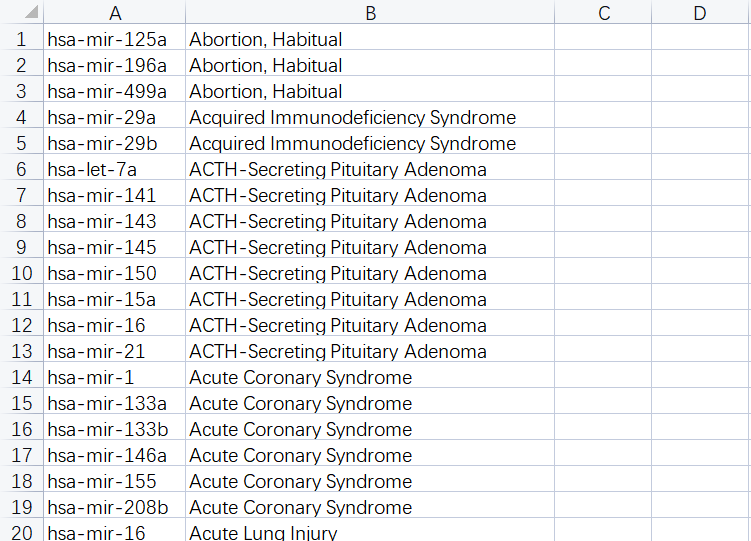
**

Figure 1.known miRNA-disease association datasets from HMDD database

**Supplementary file 2.**The lncRNA–miRNA interaction dataset was downloaded from starBase v2.0 database in January, 2015. After getting rid of duplicate interactions, 9086 lncRNA-miRNA interactions about 246 miRNAs and 1089 lncRNAs were obtained.As shown in Figure 2.

**
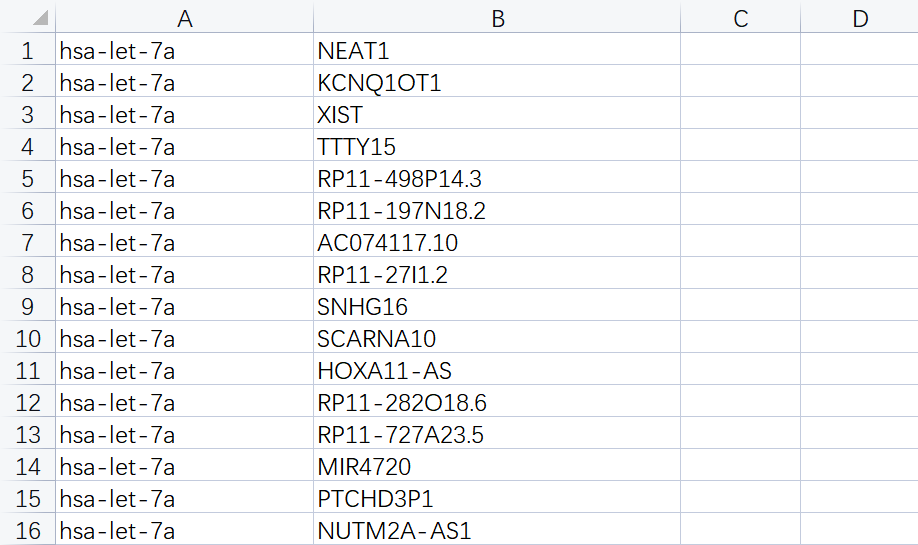
**

Figure 2.known lncRNA-miRNA association datasets from Starbase v2.0 database

**Supplementary file 3.**The lncRNA–disease interaction dataset was downloaded from MNDRv2.0 database in 2017. After getting rid of duplicate associations with different evidences and the lncRNA-disease associations involved with either diseases or lncRNAs which were not contained in the dataset used in this paper, this dataset consists of 9086 lncRNA-miRNA interactions about 373 diseases and 1089 lncRNAs were obtained.As shown in Figure 3.

**
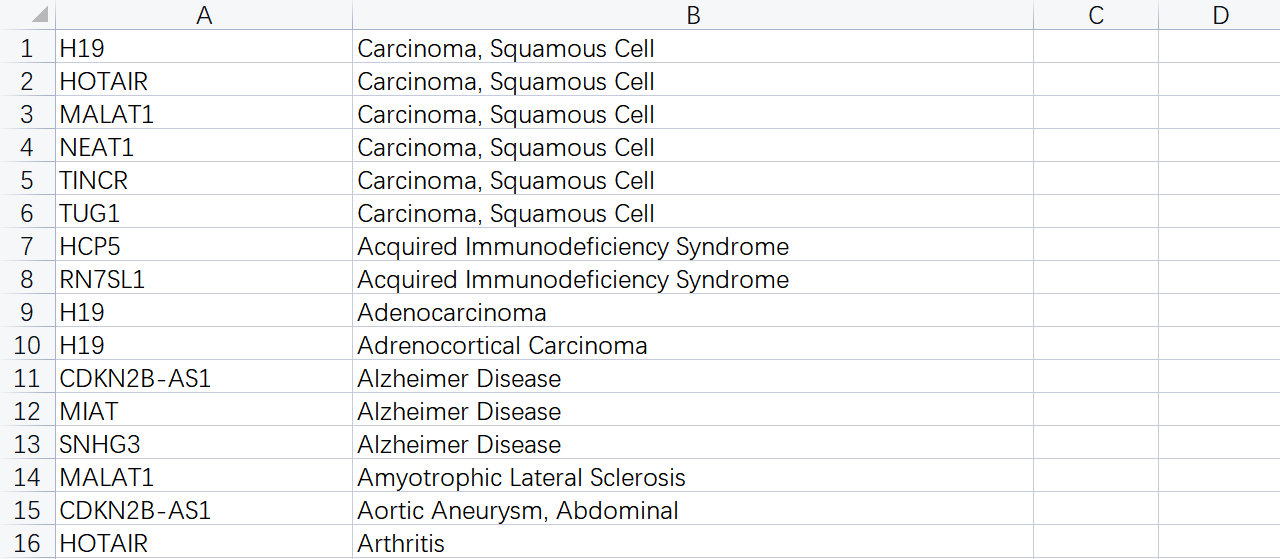
**

Figure 3.known lncRNA-disease association dataset from MNDRv2.0 database
